# Inhibition of a live-attenuated chlamydia oral vaccine in the large intestine is dependent on CD11c-expressing cells that produce IL-23

**DOI:** 10.1038/s41598-026-51625-5

**Published:** 2026-05-09

**Authors:** Ying He, Ahmed Mohamed Abdelsalam, Yi Wu, Mariah Rodroguez, Huizhou Fan, Guangming Zhong

**Affiliations:** 1 Department of Microbiology, Immunology and Molecular Genetics, University of Texas Health Science Center at San Antonio, 7703 Floyd Curl Drive, San Antonio, TX 78229 USA; 2 Department of Physiology, Robert Wood Johnson Medical School Research Tower, 675 Hoes Lane, Room 400, Piscataway, NJ 08854 USA; 3 Present Address: Department of Infectious Diseases, The 2nd Xiangya Hospital of Central South University, 139 Renmin Middle Rd, Changsha, 410011 Hunan China

**Keywords:** Chlamydia oral vaccine, IntrOv, Bone marrow-derived dendritic cells, IL-23, Large intestine, Immunology, Microbiology

## Abstract

The live-attenuated oral vaccine intrOv is an IFNγ-susceptible mutant of *C. muridarum* that is rapidly cleared from the large intestine by IFNγ-producing group 3 innate lymphoid cells (IFNγ^+^ILC3s), while the wild-type *C. muridarum* maintains long-lasting colonization. Although we have previously shown that IL-23 receptor signaling is essential for IFNγ^+^ILC3s to clear intrOv, the cellular source of IL-23 remains unknown. In the current study, we found that intrOv induces IL-23 in the large intestine, which correlates with intrOv inhibition. Mice deficient in IL-23p19 fail to inhibit intrOv, demonstrating the necessity for IL-23 to promote the inhibition of intrOv. To identify the responsible IL-23 producers, transgenic mice expressing CD11c promoter-driven diphtherial toxin receptor are used to deplete dendritic cells. We found that depletion of CD11c-expressing cells significantly reduces IL-23 and IFNγ, allowing intrOv to grow, while wild-type CD11c-expressing cells are sufficient to rescue IL-23p19-deficient mice to inhibit intrOv in the large intestine. Thus, we have demonstrated that CD11c-expressing cells inhibit the live-attenuated chlamydia oral vaccine intrOv via an IL-23-dependent mechanism, providing novel information to improve the safety and efficacy of intrOv and to reveal the intricate interactions of an obligate intracellular bacterium with host mucosal tissues.

**Supplementary Information:**

The online version contains supplementary material available at 10.1038/s41598-026-51625-5.

## Introduction

*Chlamydia trachomatis* is an obligate intracellular bacterium^1^, and sexually transmitted infections of the genital tract caused by *C. trachomatis* impose a significant health burden^2^. *C. trachomatis* is also detected in the gastrointestinal (GI) tract^3–6^. The mouse-adapted *Chlamydia muridarum* has been used to reveal chlamydial and host factors that impact chlamydial pathogenicity^7–10^, and also uncover a complex relationship between the GI and genital chlamydia. When chlamydial infection occurs in the genital tract first, the genital chlamydia spreads to the GI tract in addition to ascending to the upper genital tract. The genital tract-originated GI chlamydial colonization may promote the pathogenicity of the genital chlamydia by inducing chlamydia-specific profibrotic CD8^+^ T cells^11–13^. In contrast, if the GI tract of a naïve mouse is first exposed to chlamydia, the mouse becomes immune to subsequent chlamydial infections in extra-gut tissues^14,15^, which has inspired the development of oral vaccines against chlamydial infection in the genital tract^16–18^.

There is an urgent need to develop a human vaccine to prevent *C. trachomatis* infection. However, efforts to develop a subunit vaccine, motivated by the failure of the whole *C. trachomatis*-based trachoma vaccine trials^19–23^, have produced no licensed vaccines^24–28^. The recent success in using modified *C. trachomatis* whole organisms to induce transmucosal protective immunity in the female genital tract has rekindled interest in developing a whole-cell-based chlamydia vaccine^29^. Various genital tract pathogenicity-attenuated *C. muridarum* mutants have been identified^30–35^. Oral immunization with some of these mutants induced protection against subsequent wild-type chlamydial infections in the genital tract or airway^16–18^. The intracellular Oral vaccine vector (intrOv) mutant is being developed as an oral vaccine to induce heterotypic protection against *C. trachomatis* in the genital tract^36^. IntrOv is rapidly cleared from the GI tract by IFNγ-producing group 3 innate lymphoid cells or IFNγ^+^ILC3s^37–41^. The induction of IFNγ^+^ILC3s is dependent on IL-23 receptor (IL-23R) signaling, as mice deficient in IL-23R failed to recruit IFNγ^+^ILC3s, allowing intrOv to grow^42^. However, it remains unclear whether intrOv induces IL-23 in the large intestine and which cell types are responsible for producing it.

The current study aims to test the hypothesis that, in response to intrOv inoculation, dendritic cells produce IL-23 to recruit and activate effector IFNγ^+^ILC3s, thereby inhibiting intrOv in the large intestine. We have found that intrOv induces IL-23 in the large intestine, which correlates with the inhibition of intrOv, whereas IL-23-deficient mice allow intrOv to continuously colonize the large intestine. Furthermore, CD11c-expressing cells or dendritic cells are crucial for intrOv induction of IL-23 and IFNγ and inhibition of intrOv, as depletion of CD11c-expressing cells significantly reduces IL-23 and IFNγ and increases intrOv colonization. More importantly, bone marrow-derived CD11c-expressing cells are sufficient to rescue IL-23-deficient mice to inhibit intrOv growth. Thus, we have provided experimental evidence demonstrating a critical role for CD11c-expressing cells in inhibiting the live-attenuated chlamydia vaccine intrOv in the large intestine via an IL-23 signaling-dependent mechanism. The information is essential for improving the safety and efficacy of intrOv as an oral vaccine and revealing the intricate interactions between an obligate intracellular bacterium and mammalian mucosal tissues.

## Materials and methods

### Chlamydia organisms

The *Chlamydia muridarum* mutant clone G28.51.1^32,33^ was used in the current study. This clone is designated an intracellular oral vaccine vector, or intrOv^43^, as it lacks pathogenicity in the genital tract but induces transmucosal protection^16^. IntrOv contains a glutamine (Q) to glutamic acid (E) substitutional mutation at the 117th position of the hypothetical protein TC0237 (TC0237Q117E) and a deletion mutation in the *tc0668 gene*, converting the 216th glycine (G) codon into a premature stop codon (TC0668G216*). The double mutations make intrOv susceptible to IFNγ, and intrOv is cleared from the large intestine by IFNγ-producing group 3 innate lymphoid cells or IFNγ^+^ILC3s^37–40,42^. The intrOv organisms were grown in HeLa cells (human cervical carcinoma epithelial cells; ATCC# CCL-2), and a density-gradient purification protocol was used to purify the elementary bodies (EBs) of intrOv as described previously^44^. The purified EBs were stored in aliquots @ -80 °C until use.

### Mouse infection and treatment

The mouse experiments were conducted in accordance with the recommendations outlined in the Guide for the Care and Use of Laboratory Animals, endorsed by the National Institutes of Health. The Protocol #20230086AR used in the current study was approved by the University of Texas Health San Antonio Institutional Animal Care and Use Committee (IACUC) under assurance ID D16-00224 (A3345-01). For intracolonic inoculation, mice were anesthetized with ~ 3% isoflurane in a jar with a clear lid until they completely lost the righting reflex. After a quick intracolon inoculation, the mouse was placed on a warmed surface in a cage to recover before returning to the experimental cage. For non-survival surgeries, at the designated time points or the conclusion of each animal experiment, all mice were euthanized by overdose of isoflurane in a jar layered with paper towels and secured with a clear cover, followed by cervical dislocation, before collecting mouse tissues and/or disposing of corpses. Prior to cervical dislocation, efforts were made to ensure that animals were unresponsive to toe stimulation.

The following 6- to 10-week-old male or female mice were used in the current study: C57BL/6J (Jax stock No: 000664, Jackson Laboratories, Inc., Bar Harbor, ME), mice deficient in IL-12p35 (IL-12p35^−/−^; Jackson stock No: 002691), IL-23p19^−/−^ (Model#TF1052, Taconic Biosciences, provided by Dr. Alexei Tumanov, University of Texas Health Science Center at San Antonio), while CD11c-DTR mice (Jackson stock No: 004509) were used for depleting CD11c-expressing or dendritic cells after injection with diphtherial toxin (DT, cat#D0564, Sigma).

All mice were inoculated intracolonically without or with intrOv EBs at a dose of 1 × 10^7^ inclusion-forming units (IFUs) per mouse as described previously^16,40,45^. Briefly, the intrOv EBs diluted in 50 µl of SPG (220 mM sucrose, 12.5 mM phosphate, 4 mM l-glutamic acid, pH 7.5) buffer were delivered to the colon using a straight ball-tipped needle (N-PK 020; Braintree Scientific, Inc., Braintree, MA). After inoculation, mice were monitored for live intrOv organisms in rectal swabs or sacrificed for titrating live organisms and cytokines in organs/tissues, including stomach (STO), various small intestine (SI) segments such as duodenum (Duo), jejunum (Jej), ileum (Ile), various large intestine (LI) segments such as cecum (Cec), colon (Col), anorectum (AR), and extra gastrointestinal (GI) organs such as spleen (Spl), liver (liv), & kidney (Kid). For some experiments, each mouse was intraperitoneally injected with DT at 100ng each on even days for 2 weeks to deplete diphtherial toxin receptor (DTR)-expressing cells.

### Titrating live chlamydial organisms from mouse swabs and tissues

To monitor live chlamydial organisms shedding from the GI tract, rectal swabs were collected in 0.5 ml of SPG buffer and vortexed with glass beads to release infectious EBs. To titrate live organisms from mouse tissues/organs, the corresponding tissues/organs indicated in individual experiments were collected (on designated days specified in the corresponding experiments) into 2 ml of SPG buffer, followed by homogenization and brief sonication. Live intrOv organisms released in the supernatants were titrated in duplicate on HeLa cell monolayers grown in 96-well plates. 50 µl of each sample, without (neat) or with serial dilutions, was used to inoculate monolayer HeLa cells. After incubation overnight, the infected HeLa cells were processed for immunofluorescence labeling of chlamydial organisms as described below. The entire culture well was counted for chlamydial inclusions when the inclusion density was low, allowing detection of a single IFU per 50 µl sample. The total number of IFUs per swab or tissue was converted into log_10_ for calculating the group mean and standard deviation. Typically, the detection limits are 10 IFUs per swab and 40 IFUs per tissue sample.

### Immunofluorescence assay

The immunofluorescence assay for visualizing and counting chlamydial inclusions in the Chlamydia-infected HeLa culture was described previously^46^. Briefly, infected HeLa cells grown on 96-well plates were fixed with paraformaldehyde (Sigma, St. Louis, MO 63178) and permeabilized with Triton X-100 (Sigma). The monolayers were labeled with a rabbit anti-chlamydial antibody (raised by immunization with *C. muridarum* EBs) and a goat anti-rabbit IgG conjugated with Cy2 (green, Jackson ImmunoResearch Laboratories, Inc) to visualize chlamydial inclusions, while a Hoechst dye (blue; Sigma) was used for labeling nuclear DNA. The labeled cell samples were viewed under an Olympus IX-80 fluorescence microscope equipped with multiple filter sets (Olympus, Melville, NY).

### Measurement of cytokines in mouse tissues using ELISA

The ELISA kits for measuring mouse IFNγ and IL-23p19 from mouse tissues were purchased from Thermo Fisher Scientific (Cat# 88-7314-88 for IFNγ and Cat# 88-7230-88 for IL-23p19, Waltham, MA). 100 µl of each colon tissue homogenized in SPG buffer as described above was mixed with a protease inhibitor cocktail (cat#78430, 100x stock, Thermo Fisher Scientific) at a final concentration of 2X. The neat homogenates were 2-fold serially diluted with PBS containing 2X inhibitor cocktail, and the serially diluted samples were applied to 96-well plates precoated with a capture antibody. IFNγ or IL-23p19 binding was detected with a detection antibody plus detection reagent. The absorbance at 450 nm was measured using a Synergy H4 microplate reader (BioTek, Winooski, VT), and results were expressed as pg/ml based on the standard curve obtained from the same plate.

### Bone marrow-derived dendritic cell preparation

Bone marrow-derived dendritic cells (BMDCs) were prepared from wild-type mice as donor cells. Briefly, bone marrow was extracted from the femur and tibia of 10-week-old C57BL/6J mice. Red blood cells were lysed with Ammonium-Chloride-Potassium buffer (Thermo Fisher Scientific) for 10 min on ice, followed by two washes with 1X PBS. The cells were incubated in Roswell Park Memorial Institute (RPMI) 1640 medium (Gibco, Thermo Fisher Scientific, Inc., Waltham, MA) supplemented with 10% fetal bovine serum (FBS, Corning Inc.), 20 ng/ml mouse GM-CSF (Gibco), and 10 ng/ml mouse IL-4 (Gibco). After 7 days, the cells were harvested, and BMDCs were sorter-purified as Live CD11c+ cells.

### Flow cytometry analysis and sorting

To monitor CD11c+ cells in the spleen and blood, single-cell suspensions were prepared from the spleen & blood (days 0, 7, & 14). 50 µl of fresh blood was incubated in Ammonium-Chloride-Potassium buffer (ACK, A1049201, Thermo Fisher Scientific, Waltham, MA) on ice for 15 min. While the spleen was gently homogenized through a 70 μm strainer (Corning Inc., Austin, TX), followed by incubation for 10 min in ACK buffer (Thermo Fisher Scientific). The cells were washed twice with 1X PBS for flow cytometry analysis. Spleen & blood cells were labeled with Rat anti-mouse CD16/32 Ab (to block nonspecific binding to Fc receptors, clone: 2.4G2, cat#: BE0307, Bio X Cell, West Lebanon, NH), rat anti-mouse CD45 Ab (conjugated with BUV737, clone: 30-F11, cat#: 367-0451-82, Thermo Fisher Scientific), and stained with Viability-Dye (conjugated with eFluor 506, cat#: 65-0866-14, Thermo Fisher Scientific) to exclude dead cells., while the hamster anti-mouse CD11c antibody (conjugated with APC, clone#HL3, cat#550261, Waters Bioscience) was used to identify dendritic cells from peripheral blood and spleen samples. The antibody staining was permitted for 30 min at 4 °C. BD LSR II Flow Cytometer (Waters Bioscience) was used to analyze the samples.

To sort Bone Marrow Derived DCs into CD11c + DCs and CD11c- non-DCs as donor cells, the harvested cells were labeled with Rat anti-mouse CD16/32 Ab (to block nonspecific binding to Fc receptors, clone: 2.4G2, cat#: BE0307, Bio X Cell, West Lebanon, NH), hamster anti-mouse CD11c Ab (conjugated with APC-Cy7, clone: HL3, cat#: 561241, Waters Biosciences), and stained with Viability-Dye (conjugated with eFluor 506, cat#: 65-0866-14, Thermo Fisher Scientific). After excluding dead cells, CD11c+ cells were sorted as DCs, while CD11c- cells were sorted as non-DCs as control donor cells using the BD FACSDiscover™ S8 Cell Sorter (Waters Biosciences) (supplementary Fig. 1).

### Adoptive transfer

The transfer was performed once via retro-orbital injection, with 1 × 10^6^ cells, as indicated in the individual experiments. The transfer was performed one day before the intracolonic inoculation of recipient mice (IL23p19^−/−^) with intrOv. Following the intracolonic inoculation, live intrOv organisms were monitored in rectal swabs and tissues.

### Statistics

The number of live organisms in IFUs at individual data points or over a time course was compared using the Wilcoxon rank-sum test. Area-under-the-curve or AUC was used for comparing the time course or clusters of tissue sample data. When multiple groups were included in an experiment, ANOVA was first used to determine whether there was a significant overall difference among the groups. Only when *p* < 0.05 (ANOVA), were the differences between every two groups further analyzed using Wilcoxon.

## Results

### IL-23 is required for inhibiting intrOv in the large intestine

**Fig. 1:**
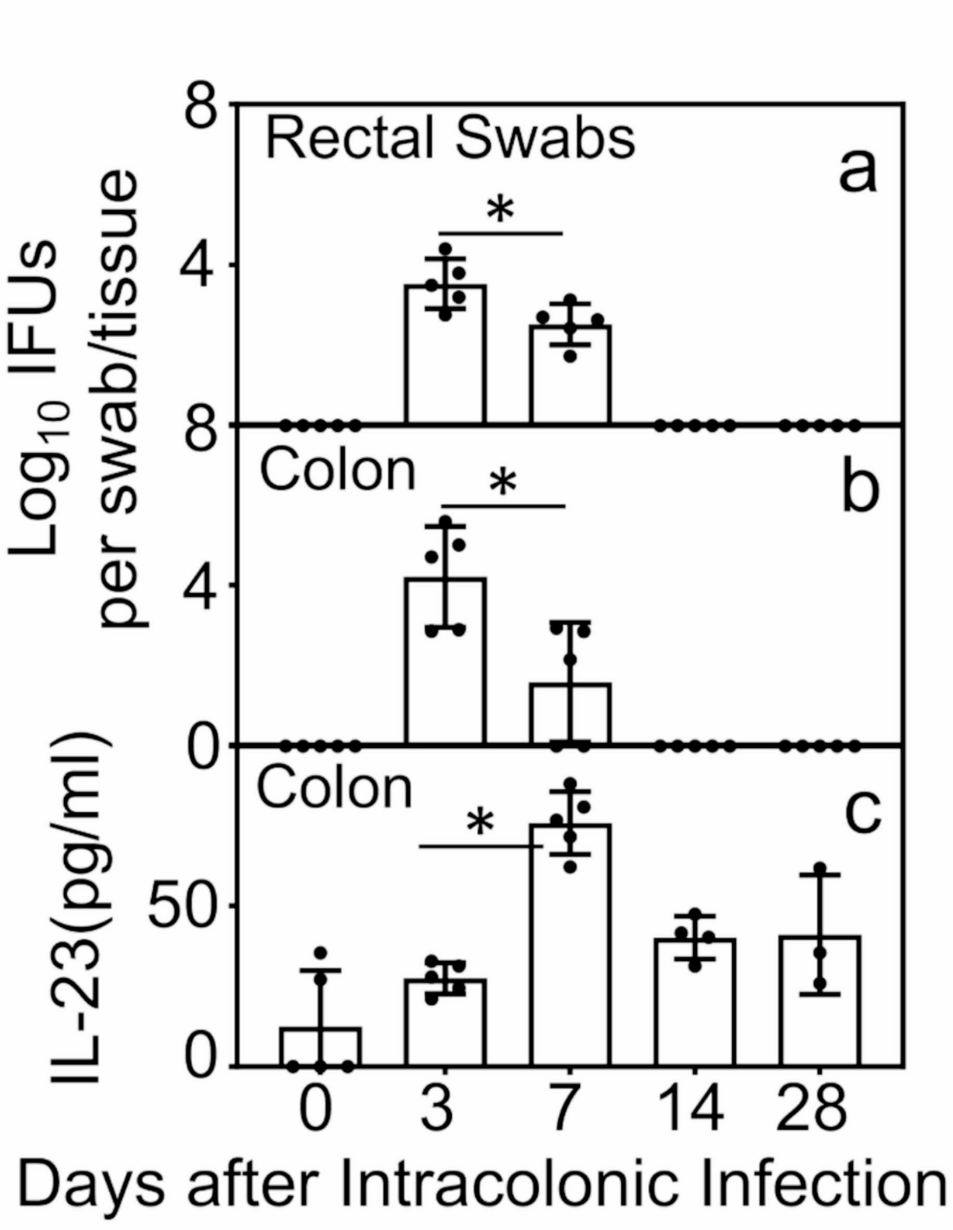
IntrOv induction of IL-23 in the large intestine. Mice intracolonically inoculated with 1 × 10^7^ inclusion-forming units (IFUs) of the attenuated oral vaccine strain intrOv were monitored for live intrOv organisms from rectal swabs (panel a, *n* = 5) or colon tissues (b, *n* = 5) and IL-23 cytokine levels in the colon tissues (c, *n* = 5) on different days post-inoculation (X-axis). Note that IL-23 peaked on day 7, correlating with the reduction in the live intrOv recoveries. The data came from 2 to 3 independent experiments. *p < 0.05, 2-tailed Wilcoxon.

**Fig. 2:**
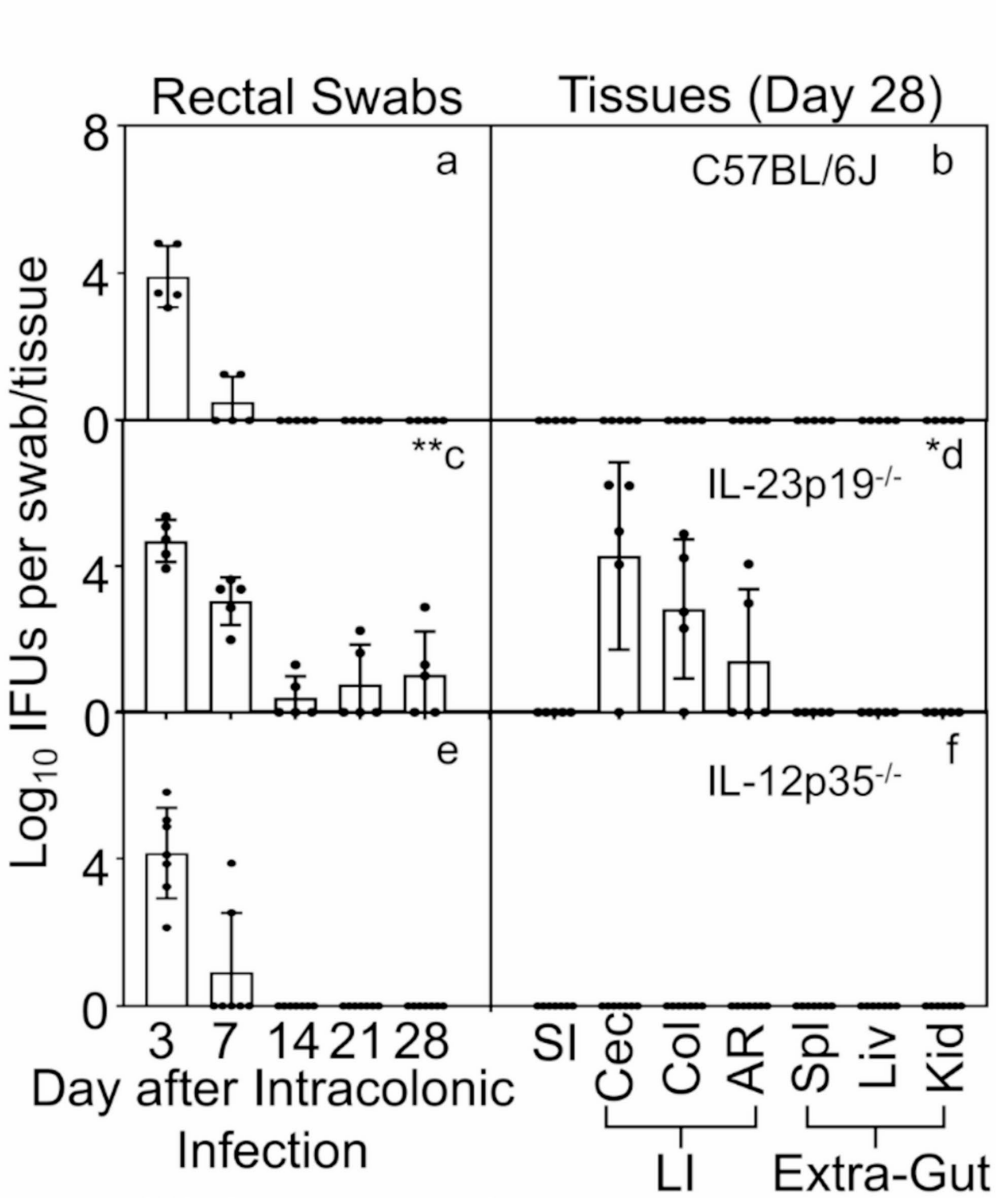
IntrOv growth in the large intestine of mice deficient in IL-23p19 or IL-12p35. Mice without (C57BL/6J, panels a & b, *n* = 5) or with deficiency in IL-23p19 (IL-23p19^−/−^, c & d, *n* = 5) or IL-12p35 (IL-12p35^−/−^, e & f, *n* = 7) were intracolonically inoculated with intrOv and monitored for live intrOv from rectal swabs (a, c & e) or tissues (b, d & f), including small intestine (SI), the large intestinal tissues (LI) cecum (Cec), colon (Col), annal rectum (AR), and extra-gut tissues spleen (Spl), liver (Liv), and kidney (Kid), as listed along the X-axis. Note that IL-23p19^−/−^ but not IL-12p35^−/−^ allowed intrOv to shed live organisms from and persist in the large intestine; The data came from 2 to 3 experiments. *p < 0.05 & **p < 0.01 (2-tailed Wilcoxon), Area Under Curves (AUCs) from panels c and e were compared against that in panel a, while those from panels d & f against that of panel b.

We previously determined that the orally inoculated intrOv was cleared from the GI tract by IFNγ^+^ILC3s in the large intestine^38,39^. Using an intracolonic inoculation model, we further found that the IFNγ^+^ILC3-mediated clearance of intrOv is dependent on IL-23R signaling^40^. In the current study, we monitored IL-23 production in colon tissue following intracolonic inoculation with intrOv (Fig. 1). IL-23 was significantly induced and peaked on day 7 after intrOv inoculation, which coincided with a significant reduction in the infectious yield of intrOv recovered from colon tissue. By day 14 after intracolonic inoculation, intrOv was completely cleared from shedding infectious organisms in the rectal swabs and persisting in the tissue. These results correlated the induction of IL-23 by intrOv with the inhibition of intrOv. More importantly, as shown in Fig. 2, mice deficient in IL-23p19 allowed intrOv to continuously shed live intrOv in rectal swabs and to persist in colonic tissue while mice deficient in IL-12p35 failed to do so. These observations have demonstrated a critical role for IL-23, but not IL-12, in inhibiting intrOv.

### The induction of IL-23 by intrOv in the large intestine is dependent on CD11c-expressing dendritic cells

**Fig. 3:**
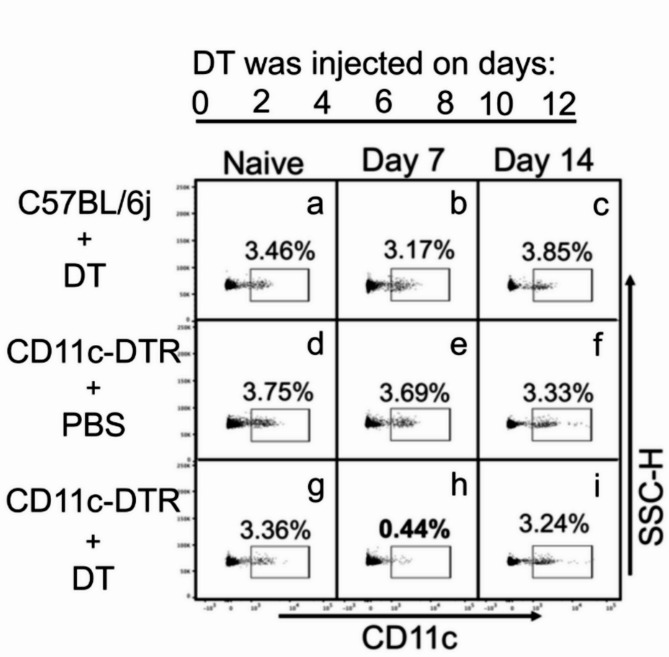
Monitoring CD11c+ cells in the peripheral blood following diphtherial toxin (DT) injection. C57BL/6J (panels a-c) or diphtherial toxin receptor (DTR) transgenic mice (CD11c-DTR, d-i) were intraperitoneally injected without (PBS, d-f) or with DT (a-c & g-i) at 100ng each starting on day 0 and thereafter on even days for 2 weeks to deplete the diphtherial toxin receptor (DTR)-expressing cells. Peripheral blood cells were collected before DT injection (naïve) and on days 7 and 14 after DT injection, respectively, to monitor the frequency of CD11c+ cells among CD45 + cells in the circulation. On day 7, a significant reduction in CD11c+ cells was observed in CD11c-DTR mice that received DT but not CD11c-DTR mice that received PBS buffer only or C57BL/6J mice that received DT, indicating a specific deletion of CD11c+ cells. By day 14, the CD11c-DTR + DT mice had restored normal levels of CD11c+ cells in the peripheral blood. Only representative flow charts from each group were shown, and the results were consistent among all mice in the same group.

**Fig. 4:**
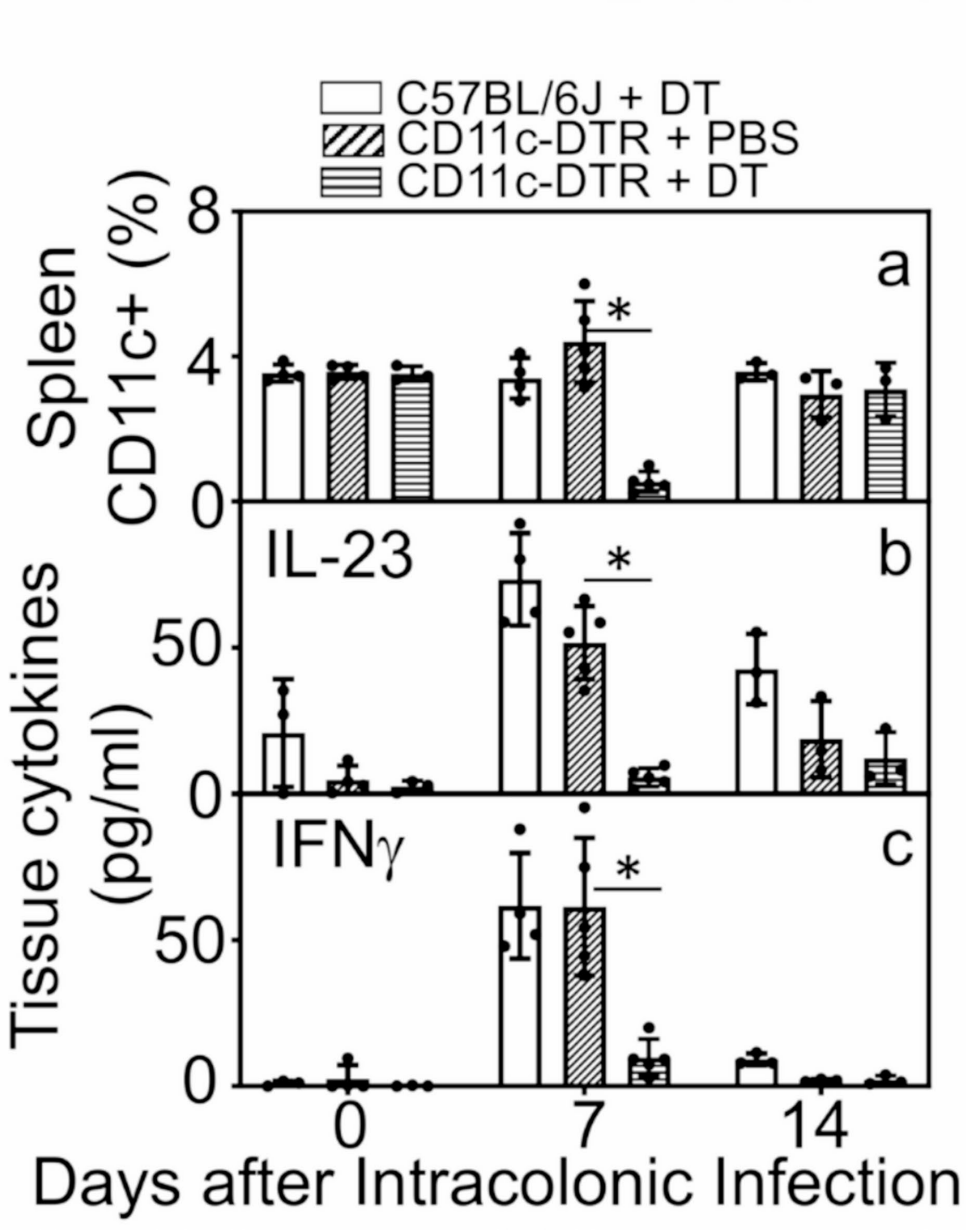
IntrOv induction of IL-23 and IFNγ in the large intestine following depletion of CD11c+ cells. Wild-type (open bar, C57BL/6J, *n* = 5) or transgenic (expressing diphtherial toxin receptor under the control by the mouse CD11c promoter, CD11c-DTR) mice treated with the phosphate-buffered solution (PBS, slanted hatched bar, *n* = 5) or diphtherial toxin (DT, horizontal hatched bar, *n* = 5) were intracolonically inoculated with intrOv and monitored for CD11c+ cells in the spleen (a), IL-23 (b) and IFNγ (c) in the colon tissue on days 7 & 14 after the intracolonic inoculation. Samples from uninfected mice were used to establish the baseline levels of IL-23 and IFN-γ (day 0). Note that the DT-induced depletion of DCs correlated with the reduction in both IL-23 and IFNγ in the large intestine. The data came from 2 independent experiments. *p < 0.05, 2-tailed Wilcoxon.

To determine the cellular basis of IL-23 production during intrOv colonization in the large intestine, we evaluated the role of DCs, as myeloid cells, particularly activated DCs, are major producers of IL-23 during microbial infections^47,48^. When mice expressing the diphtherial toxin receptor (DTR) under the control of the CD11c promoter (CD11c-DTR) were injected with a diphtherial toxin (DT) on alternate days, the CD11c+ cells were significantly depleted during the 1st week but recovered afterward (Fig. 3), which is consistent with the known limitation of this approach^49^. Nevertheless, the depletion of DCs during the 1st week was sufficient to significantly reduce IL-23 and IFNγ production in the colon on day 7 after intrOv inoculation (Fig. 4).

### Depletion of CD11c-expressing DCs partially rescues the growth of intrOv in the large intestine

**Fig. 5:**
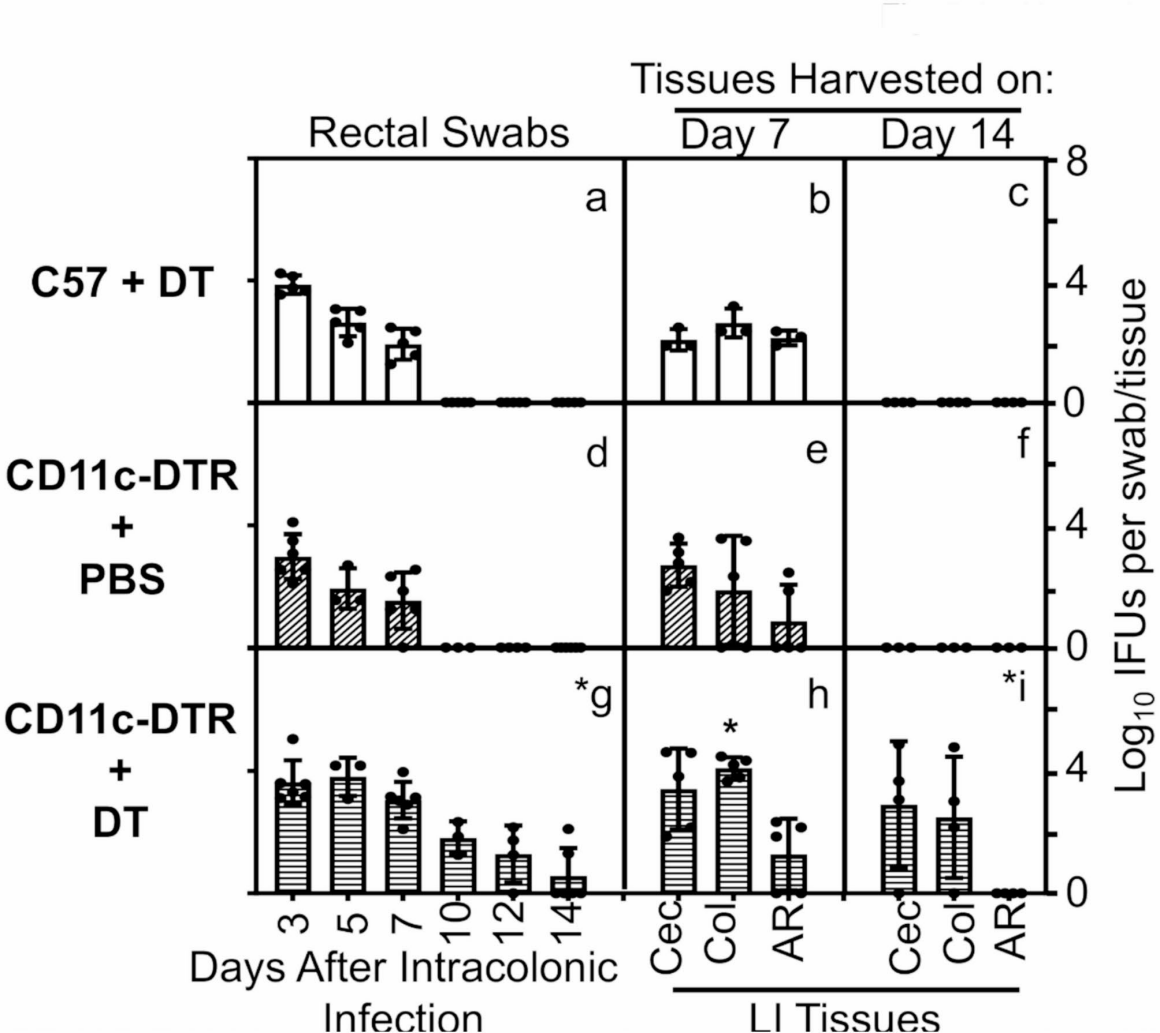
IntrOv growth in the large intestine following depletion of CD11c+ cells. Wild-type treated with DT (panels a-c, open bar, *n* = 5) or transgenic CD11c-DTR mice treated with PBS (d-f, slanted hatched bar, *n* = 5) or DT (g-i, horizontal hatched bar, *n* = 5) were intracolonically inoculated with intrOv and monitored for live intrOv organisms from rectal swabs along the time course (a, d & g) or selected tissues on day 7 (b, e, & h) or 14 (c, f, & i). The data came from 2 independent experiments. *p < 0.05, 2-tailed Wilcoxon (between CD11c-DTR mice with or without DT in terms of single data point or AUCs).

We further monitored the growth of intrOv in the large intestine following CD11c-expressing DC depletion (Fig. 5). IntrOv grew to a limited extent in the large intestine of C57 mice receiving DT or of CD11c-DTR mice receiving PBS alone. Live intrOv organisms were recovered from the rectal swabs collected during the 1st 7 days and the large intestine tissues on day 7, respectively. No live organisms were detected in these mice beyond day 7. The growth pattern is consistent with previous findings that intrOv can only transiently colonize the large intestine^38,39^. On the contrary, the colonization of intrOv in the large intestine persisted through day 14 in CD11c-DTR mice receiving DT. Thus, depletion of DCs significantly extended the colonization of intrOv, demonstrating a critical role of DCs in inhibiting intrOv in the large intestine.

### CD11c-expressing bone marrow-derived cells are sufficient to rescue IL-23p19^−/−^ mice to inhibit the growth of intrOv in the large intestine

**Fig. 6:**
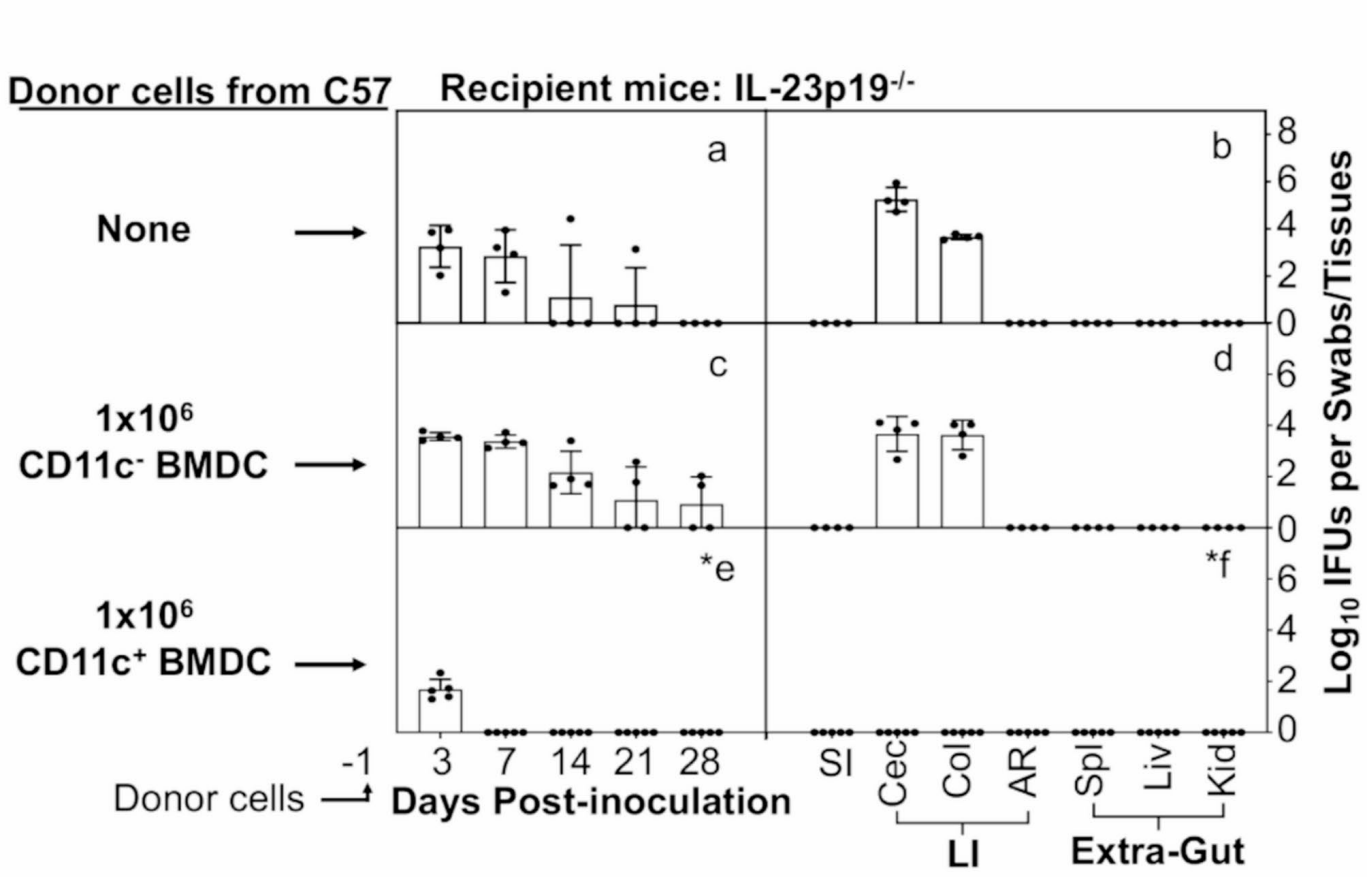
IntrOv growth in the large intestine of IL-23p19-deficient mice following adoptive transfer with wild-type CD11c+ cells. Bone marrow-derived dendritic cells (BMDCs) prepared from wild-type C57 mice were sorted into CD11c^+^ and CD11c^−^ donor cells (left) for adoptive transfer to groups of IL-23p19^−/−^ recipient mice (*n* = 4/group) one day before intracolonial inoculation with intrOv. Following intracolonic inoculation, rectal swabs were taken from the recipient mice on days 3, 7, and weekly thereafter (X-axis). On day 28, mice were euthanized to titrate live intrOv from different tissues listed along the X-axis. The results were expressed as log10 IFUs per swab or tissue (Y-axis). The data came from two independent experiments. *p < 0.05, two-tailed Wilcoxon, AUCs between the CD11c+ donor recipient group (panel c) and the recipient groups receiving no donor cells (a) or CD11c- donor cells (b). Note that intrOv’s shedding from and persistence in the large intestine is prevented by CD11c + but not CD11c- donor cells.

We further evaluated whether DCs are sufficient for promoting the inhibition of intrOv in the large intestine. Although intrOv can only grow to a limited extent in the large intestine of C57 mice, it can achieve extensive growth in IL-23p19-deficient mice, as demonstrated above. We then used the IL-23p19^−/−^ mice as recipients to address the sufficiency issue (Fig. 6). As expected, live intrOv was recovered from the large intestine tissues of the IL-23p19^−/−^ mice on day 28. However, adoptive transfer of CD11c+ BMDCs efficiently cleared intrOv from both shedding into the lumen and from persisting in the tissue, as live intrOv was no longer detected on day 7, and no live intrOv was detected in the tissue on day 28. Thus, CD11c + DCs are sufficient to rescue the susceptible IL-23p19^−/−^ mice to efficiently inhibit intrOv. As a control, a similar adoptive transfer of CD11c-negative cells sorted from the same BMDC preparation failed to significantly alter colonization of intrOv in IL-23p19^−/−^ mice. Thus, rescue depends on CD11c+ cells.

## Discussion

Extensive efforts are ongoing to develop an effective and safe human chlamydia vaccine^50^. Although a subunit vaccine was evaluated in a phase I clinical trial 7 years ago^27^, there is still no licensed human chlamydia vaccine. In addition to efforts to identify and evaluate chlamydial antigens as subunit vaccines^24,26,51,52^, parallel efforts also include the development of whole chlamydial cell-based vaccines^16,17,53^, which have been encouraged by the understanding of the mechanisms by which formalin-killed chlamydial cell-based vaccines failed to induce durable protection and even exacerbated inflammatory pathologies upon subsequent exposure to chlamydial infection^29^. We have been developing a live-attenuated oral chlamydia against *C. trachomatis* infection, which is designated as intrOv^16^. IntrOv is a genital tract pathogenicity- attenuated *C. muridarum* mutant^33^, and oral immunization with intrOv induces transmucosal protection in the genital tract^16^. On one hand, we are providing IND-enabling data for moving intrOv to clinical trials by demonstrating the intrOv induction of heterotypic protection against *C. trachomatis* infection in the genital tract of nonhuman primates, and on the other, we are continuously investigating the mechanisms of immune responses induced by intrOv in the GI tract, as these mechanisms are essential for both improving the efficacy and safety of intrOv as an oral vaccine. Our prior work has shown that intrOv is cleared from the large intestine by IFNγ^+^ILC3s^38,40^, which depend on IL-23 signaling^42^. In the current study, we have identified dendritic cells as the IL-23 producers to promote the inhibition of intrOv in the large intestine. First, intrOv induces IL-23 in the large intestine, and IL-23 production correlates with reduced intrOv colonization. Second, mice deficient in IL-23p19 but not IL-12p35 allow intrOv to continuously colonize the large intestine, indicating the necessity of IL-23 in inhibiting intrOv. Third, dendritic cells are crucial for IL-23 and IFNγ production and for inhibiting intrOv, as their depletion significantly reduces IL-23/ IFNγ and promotes intrOv colonization in the large intestine. Finally, adoptive transfer of wild-type dendritic cells restores IL-23p19-deficient mice to inhibit intrOv, demonstrating the sufficiency of dendritic cells in promoting intrOv inhibition.

IL-23 can be produced by myeloid cells^47,48^, and intestinal infection with bacteria such as *Citrobacter rodentium* has been shown to induce DCs to produce IL-23^54^. To probe the cellular basis of IL-23 production following intracolonic inoculation with intrOv, we used transgenic mice that express diphtherial toxin receptor under CD11c promoter to deplete dendritic cells^55^. Depletion of dendritic cells significantly reduced IL-23 and IFNγ, which was accompanied by increased colonization of intrOv in the large intestine. Thus, we have demonstrated that dendritic cells are crucial in both the production of IL-23 and IFNγ and the inhibition of intrOv. This finding is novel, as the cellular basis of IL-23 production during chlamydial infection was unknown prior to the current study. The next question is to further characterize the responsible dendritic cells, including identifying their subsets and determining how they are activated by chlamydial colonization, which is underway.

The finding that intrOv induces CD11c-expressing DCs to produce IL-23 in the large intestine is significant. First, the DCs-IL-23 axis contributes to the safety of intrOv, as intrOv is being developed into a live-attenuated oral Chlamydia vaccine. The DCs-IL-23-dependent mechanism blocks infectious shedding of intrOv and prevents its persistence in the large intestine, thereby ensuring intrOv’s safety. Second, the DC-IL-23 axis may represent a novel mechanism for regulating the commensal interactions between an obligate intracellular bacterium and the large intestine, as wild-type *C. muridarum* is considered a gut commensal in mice. Finally, mechanistic information will enhance our understanding of chlamydial pathogenic mechanisms in the genital tract, as GI chlamydial organisms may directly serve as reservoirs for repeatedly infecting the genital tract^56^ and indirectly promote immunopathogenicity of genital chlamydial infection^13^.

The fact that *C. muridarum* colonizes the mouse large intestine for long periods without causing pathology suggests that *C. muridarum* may have become a commensal species in the mouse gut^14,57,58^. *C. muridarum* must have possessed the ability to evade the immunity mediated by the DC-IL-23-IFNγ^+^ILC3s axis. Microbiota confer colonization resistance against pathogens^59–61^. Maintaining basal levels of type I^62^ and type II^60^ IFNs by the microbiota has been shown to contribute to colonization resistance. We hypothesize that the wild-type *C. muridarum*-induction and evasion of the DC-IL-23-IFNγ^+^ILC3s axis may represent a mechanism adapted by *C. muridarum* to maintain a basal level of IFNγ in the GI tract. This basal level of IFNγ is sufficient for clearing intrOv but insufficient for inhibiting the colonization of wild-type *C. muridarum.*

Since intrOv is being developed into a live-attenuated oral Chlamydia vaccine, it is necessary to investigate the mechanisms by which oral intrOv induces transmucosal protection in the genital tract. In addition to ensuring the safety of intrOv, the DCs-IL-23-IFNγ^+^ILC3s axis may also promote the immunogenicity of intrOv as an oral vaccine. This is because ILC3s are a subset of RORγt+ antigen-presenting cells^63–65^, whereas DCs are professional antigen-presenting cells^66^. Both DCs and IFNγ^+^ILC3s recruited by the DC-IL-23 signaling may present intrOv epitopes to T cells. Efforts are underway to evaluate the relative contributions of DCs and IFNγ^+^ILC3s as antigen-presenting cells to the induction of transmucosal immunity by oral intrOv^16,42^.

## Supplementary Information

Below is the link to the electronic supplementary material.

Supplementary Material 1

Supplementary Material 2

## Data Availability

All raw data reported in the current manuscript will be available in Excel file as supplementary materials.
