# Supplementary material for: Inhibition of a live-attenuated chlamydia oral vaccine in the large intestine is dependent on CD11c-expressing cells that produce IL-23
Source: Sci Rep. 2026 May 9;16:21269. doi: 10.1038/s41598-026-51625-5 (PMC13346799; doi:10.1038/s41598-026-51625-5)

**Figure 1S. Gating strategy for sorting the bone marrow-derived dendritic cells (BMDCs) into CD11c^+^ and CD11c^−^ donor cells for adoptive transfer.**

Bone marrow cells were harvested from C57BL/6J mice and cultured for 6 days in the presence of GM-CSF and IL-4 to induce bone marrow-derived dendritic cells (BMDCs). The cell suspension was surface-labeled with a live dye and anti-CD11c antibody before being subjected to a flow cytometry sorter. Single live cells were sorted into CD11c + and CD11c- wells, respectively.


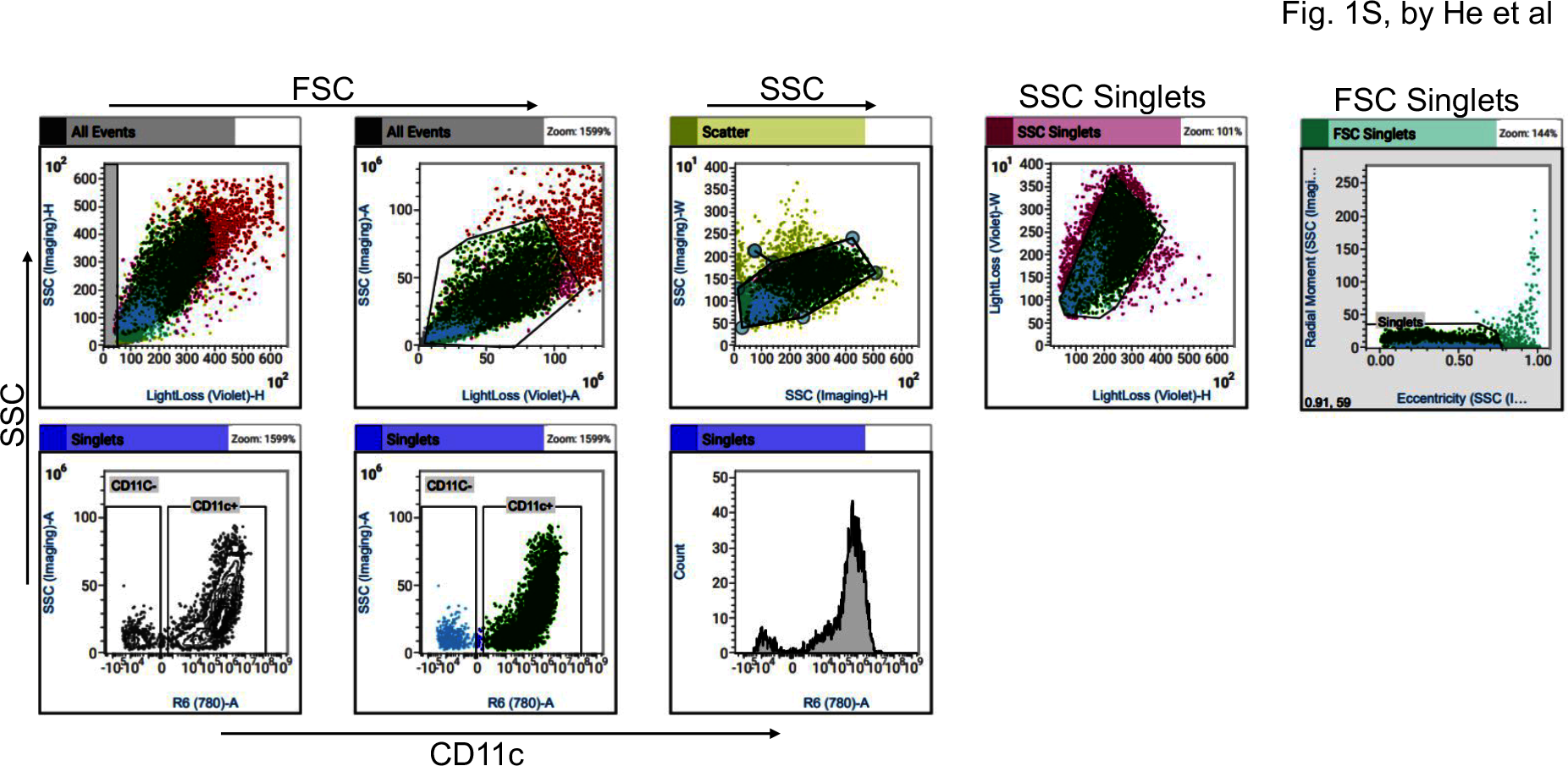

Supplement: Supplementary file 2 — Supplementary Material 2 [file 41598_2026_51625_MOESM2_ESM.docx]
